# Ocular graft-versus-host disease after allogeneic hematopoietic stem cell transplantation in a pediatric population

**DOI:** 10.1016/j.htct.2025.103823

**Published:** 2025-04-21

**Authors:** Cinthia Kim, Patricia Cabral Zacharias Serapicos, Cintia Monteiro Lustosa, Adriane da Silva Santos Ibanez, Victor Gottardello Zecchin, Lauro Augusto de Oliveira

**Affiliations:** a Department of Ophthalmology and Visual Sciences, Federal University of São Paulo (UNIFESP), São Paulo, Brazil; b Pediatric Oncology Institute, GRAACC, Federal University of São Paulo (UNIFESP), São Paulo, Brazil

**Keywords:** Graft-versus-host disease, Hematopoietic stem cell transplantation, Ocular graft-versus-host disease, Dry eye

## Abstract

**Aim:**

To determine the prevalence of ocular graft-versus-host disease after allogeneic hematopoietic stem cell transplantation and to characterize the risk factors associated with its development in a pediatric population.

**Methods:**

This retrospective chart review included 105 patients during a five-year period (2013–2017) from the Pediatric Oncology Institute (GRAACC-UNIFESP). The diagnosis of graft-versus-host disease was performed by the treating hematologist in conjunction with an ophthalmologist in accordance to National Institutes of Health (NIH) consensus criteria.

**Results:**

Systemic graft-versus-host disease occurred in 44 of 105 (41.9%) patients, predominantly in males (54.5%) whereas ocular disease was diagnosed in seven (6.7%) of the patients. All the analyzed risk factors including diagnosis, type of conditioning regimen, use of radiotherapy in conditioning, donor sex, type and source of graft, human leukocyte antigen mismatch, and sex mismatch were not statistically significantly associated with the development of ocular disease, except for age. Ocular graft-versus-host disease patients presented a higher mean age compared to patients without ocular disease (p-value = 0.015).

**Conclusion:**

Although less prevalent than in adults, ocular morbidity remains a concern in pediatric patients following allogeneic transplantation. Early diagnosis and regular ophthalmic follow-ups are recommended after the transplantation regardless of systemic graft-versus-host disease status.

## Introduction

Allogeneic hematopoietic stem cell transplantation (allo-HSCT) is an established therapeutic modality for hematological neoplasms and non-neoplastic hematological disorders. The number of patients receiving this therapy has increased as a result of improvements in the assessment of the compatibility of human leukocyte antigens (HLA) in conjunction with advances in pre- and post-transplantation therapeutic regimens, as well as better supportive care. However, the occurrence of complications is increasing due to the longer survival of patients after transplantation [1].

Graft-versus-host disease (GvHD) is a common cause of morbidity and mortality after aHSCT. It is mediated primarily by donor T lymphocytes that recognize and target host antigens leading to inflammation and fibrosis of affected tissues. This condition usually occurs in the first three years after transplantation and can affect several organs such as skin, lung, liver, gastrointestinal tract, oral mucosa, the musculoskeletal system and eyes [1,2].

According to the National Institutes of Health (NIH) consensus in 2005, GvHD can be classified as acute or chronic. The chronic form is the most common affecting 30–70% of transplanted patients [3], however there are high concordance rates between both forms as the acute form is considered a strong predictor of chronic disease. Przepiorka et al. [4] reported that patients with acute GvHD had a higher risk of chronic disease and therefore the risk factors for acute GvHD can also be applied to the chronic form [1,4].

Ocular GvHD can occur in both forms, although it is more commonly seen in the chronic form [4]. Its incidence, of between 30% and 85%, is widely variable [1]. Ocular manifestations primarily affect the cornea, conjunctiva, lacrimal glands, eyelids and meibomian glands with dry eye syndrome (DES) or keratoconjunctivitis sicca being the most common manifestation in oGvHD [5].

Major symptoms of oGvHD are irritation, burning, pain, redness, foreign body sensation, excessive tearing, light sensitivity and visual haze. Clinical findings may include acute conjunctival inflammation, conjunctival hyperemia, chemosis, pseudomembranous epitheliopathy, filaments, painful erosions, cicatricial conjunctivitis, corneal opacification, corneal ulceration, and perforation. Disease severity is variable and can negatively affect quality of life and daily life activities [1,2,6].

The risk factors for oGvHD are not well understood but it is known that history of acute and severity of chronic GvHD, female donor, peripheral blood stem cell transplantation, conditioning regimen, and HLA mismatch are associated with increased development of ocular disease [1,2,5].

This study aims to determine the frequency of oGvHD after allo-HSCT and to identify risk factors associated with its development in a population of a pediatric oncology center.

## Methods

This was a cross-sectional study based on a database review of allo-HSCT patients from January 2013 to December 2017 at the Pediatric Oncology Institute (GRAACC - Grupo de Apoio ao Adolescente e à Criança com Câncer), Federal University of São Paulo. During the study period, 108 patients underwent allo-HSCT. All medical records were reviewed, and clinical data were collected including demographic details, diagnosis, conditioning regime, source of graft, type of transplant, donor characteristics (sex, HLA matching), occurrence of acute or chronic systemic GvHD, and occurrence of oGvHD.

The diagnoses of systemic GvHD and oGvHD were performed by the treating hematologist in conjunction with an ophthalmologist in accordance to the 2005 and 2014 NIH consensus criteria (low Schirmer test values with a mean value of both eyes ≤5 mm at five minutes or a new onset of keratoconjunctivitis sicca by slit-lamp examination with mean values of 6–10 mm in the Schirmer test) [7].

The diagnoses of acute and chronic GvHD were based on clinical signs and symptoms, laboratory tests and whenever possible, on histopathologic findings of skin, oral mucosa and the gastrointestinal tract. The current consensus considers that clinical manifestations, and not the time to symptomatic onset after transplantation, determine whether the clinical syndrome of GvHD is considered acute or chronic [8].

Data were collected and presented in contingency tables. Continuous variables were compared using the Mann-Whitney test and categorical variables using the Fisher exact test. P-values of <0.05 were considered statistically significant. All analyses were achieved using the Stata v.14 computer program (College Station, Texas).

## Results

A total of 108 pediatric patients were submitted to allo-HSCT and were regularly followed up at the Pediatric Oncology Institute - GRAACC - UNIFESP during the study period. All transplants were performed by the same hematology team supervised by the same physician. Of these patients, 105 survived longer than 90 days after allo-HSCT and were included in this study. Three patients died due to disease progression a few weeks after the transplant and were therefore excluded from the analysis. Patient demographics and transplant characteristics are summarized in Table 1.

**Table 1:** Patient demographics and transplant characteristics.

Characteristic	n (%)
Total patients - n	105
Age (mean) – n (range)	9.4 (1.8–17.9)
Sex (male:female) – n (%)	63:42 (60:40)
Disease – n (%)	
ALL	38 (36)
AML	29 (28)
AA	15 (14)
Others	23 (22)
Stem cell source – n (%)	
Bone marrow	88 (84)
Peripheral blood	12 (11)
Cord blood	5 (5)
Donor sex (male:female) – n (%)	52:53 (50/50)
Donor and HLA histocompatibility – n %)	
Related identical donor	40 (38)
Mismatched related donor	10 (10)
Unrelated identical donor	35 (33)
Mismatched unrelated donor	20 (19)
Conditioning regimen (type) - n (%)	
Myeloablative	83 (79)
Reduced Intensity	22 (21)
Conditioning radiotherapy – n (%)	
Yes	54 (51)
No	51 (49)
Conditioning regimen – n (%)	
TBI + *F* + Cy	24 (23)
TBI + ETO	16 (15)
TBI + Cy	13 (12)
Bu + *M*	12 (11)
*F* + Cy + ATG	7 (7)
ALEM + *F* + *M*	7 (7)
Bu + *M* + ATG	6 (6)
Bu + Cy + *M* + ATG	4 (4)
Bu + Cy +*M*	4 (4)
Others	12 (11)
GvHD prophylaxis – n (%)	
CsA + MTX	40 (38)
CsA	31 (29)
Cy+ MMF + TAC	14 (13)
MTX + TAC	9 (9)
CsA + MMF	8 (8)
CsA + MMF + Cy	1 (1)
CsA	1 (1)
MTX	1 (1)

ALL, acute lymphoblastic leukemia; AML, acute myeloid leukemia; AA, aplastic anemia; HLA, human leukocyte antigen; TBI, total body irradiation; FLU, fludarabine; CY, Cyclophosphamide; ETO, etoposide (Etoposide); BU, busulfan; M, melphalan; ATG, anti-thymocyte globulin; ALEM, alemtuzumab; CsA, cyclosporine (Cyclosporine capsules); MTX, methotrexate (Methotrexate); MMF, mycophenolate mofetil (CellCept); TAC, tacrolimus; GvHD, graft-versus-host disease.

The mean age of this population was 9.4 (standard deviation [SD]: 4.9) years and 63 (60%) were male. The most common indications for allo-HSCT were acute lymphoblastic leukemia (*n* = 38; 36.2%), followed by acute myeloid leukemia (*n* = 29; 27.6%), aplastic anemia (*n* = 15; 14.3%), and other reasons (*n* = 23; 21.9%).

Systemic GvHD occurred in 44 (41.9%) individuals in this study population with 24 (54.5%) being male. The mean age of those with systemic GvHD was 8.8 (SD: 5.3) years compared to those without GvHD (9.5; SD: 4.6 years; p-value = 0.491). Potential risk factors associated with the development of systemic GvHD were analyzed in this population. None of the risk factors analyzed were significantly associated with the development of systemic GvHD (Table 2).

**Table 2:** Risk factors for systemic graft-versus-host disease (*n* = 105).

Risk factor	Systemic GvHD		p-value
	No (*n* = 61)	Yes (*n* = 44)	
Age, years – mean (SD)	9.5 (4.6)	8.8 (5.3)	0.491
Sex – n (%)			0.333
Male	39 (63.9)	24 (54.5)	
Female	22 (36.1)	20 (45.4)	
Diagnosis – n (%)			0.852
AAL	22 (36.1)	16 (36.3)	
AML	17 (27.9)	12 (27.3)	
AA	10 (16.4)	5 (11.4)	
Others	12 (19.6)	11 (25.0)	
Conditioning regiment – n (%)			0.704
Myeloablative	49 (80.3)	34 (77.3)	
Reduced intensity	12 (19.7)	10 (22.7)	
Radiotherapy conditioning – n (%)			0.587
No	31 (50.8)	20 (45.4)	
Yes	30 (49.2)	24 (54.6)	
Donor sex – n (%)			0.632
Male	29 (47.5)	23 (52.3)	
Female	32 (52.5)	21 (47.7)	
Source of graft – n (%)			0.862
Bone marrow	49 (87.5)	39 (88.6)	
Peripheral blood	7 (12.5)	5 (11.4)	
Type of transplant – n (%)			0.242
Related donor	29 (47.5)	26 (59.1)	
Unrelated donor	32 (52.5)	18 (40.9)	
HLA mismatch – n (%)			0.724
No	41 (67.2)	31 (70.4)	
Yes	20 (32.8)	13 (29.6)	
Sex mismatch – n (%)			0.912
No	27 (44.3)	19 (43.2)	
Yes	34 (55.7)	25 (56.8)	

GvHD, graft-versus-host disease; SD, standard deviation; ALL, acute lymphoblastic leukemia; AML, acute myeloid leukemia; AA, aplastic anemia.

A total of 40 (38.1%) individuals presented acute GvHD; the organs involved were skin in 36 (90.0%), liver in three (7.5%), and the gastrointestinal tract in eight (20.0%). Chronic GvHD occurred in 33 patients (31.4%); the organs involved were skin (*n* = 16; 48.5%), liver (*n* = 7; 21.2%), lungs (*n* = 6; 18.2%), the gastrointestinal tract (*n* = 4, 12.1%), mouth (*n* = 11; 33.3%), and eyes (*n* = 7; 21.2%). Eighteen out of 33 subjects (54.5%) had chronic GvHD following acute GvHD, while 15 (45.5%) had chronic GvHD occurring *de novo*.

Ocular GvHD was diagnosed in seven patients with GvHD (7/105; 6.7%). There were no cases of acute ocular disease. The characteristics of these patients are summarized in Table 3.

**Table 3:** Ocular graft-versus-host disease patient characteristics.

Patient	1	2	3	4	5	6	7
Sex	M	F	M	M	F	M	M
Age	18	15	10	12	11	13	16
Diagnosis	AA	ALL	AML	AML	AML	AML	ALL
Conditioning regimen	Reduced intensity	Myeloablative	Myeloablative	Myeloablative	Myeloablative	Myeloablative	Myeloablative
Conditioning radiotherapy	No	Yes	No	No	No	No	Yes
Source of graft	Bone marrow	Bone marrow	Bone marrow	Bone marrow	Bone marrow	Bone marrow	Bone marrow
Donor and HLA histocompatibility	Related identical	Unrelated identical	Related identical	Related identical	Related identical	Unrelated identical	Unrelated identical
Onset of GvHD (days)	225	69	260	364	378	366	121
Slit lamp examination	Corneal Punctate epithelial erosions	Corneal Punctate epithelial erosions	Corneal punctate epithelial erosions	Corneal punctate epithelial erosions	Conjunctival hyperemia, corneal punctate epithelial erosions	Conjunctival hyperemia, Corneal punctate epithelial erosions	Corneal punctate epithelial erosions
Schirmer`s test (mean value in mm)	5.5	4.5	5	5	0	7.5	2.5
Eye treatment	lubricant eye drops+ topical cyclosporine (Cyclosporine capsules)	lubricant eye drops+ topical cyclosporine (Cyclosporine capsules)	lubricant eye drops+ topical cyclosporine (Cyclosporine capsules)	lubricant eye drops+ topical cyclosporine (Cyclosporine capsules)	lubricant eye drops+ topical tacrolimus and steroid	lubricant eye drops+ topical steroid	lubricant eye drops
Immunosuppression after GVDH	Yes (PDN + CsA)	Yes (PDN + TAC)	Yes (PDN + CsA + IM)	Yes (PDN + CsA)	Yes (PDN + CsA + MTX + IM)	No	Yes (PDN + CsA + MFM + TAC)
Other organs involved	none	skin	skin, mouth, lungs	none	skin, lungs	skin	skin, mouth, liver, osteoarticular

M, male; F, female; AA, aplastic anemia; ALL, acute lymphoblastic leukemia; AML, acute myeloid leukemia; PDN, prednisone; CsA, cyclosporine (Cyclosporine capsules) TAC, tacrolimus; IM, imatinib; MTX, methotrexate (Methotrexate); MMF, mycophenolate mofetil (CellCept).

Ocular GvHD occurred in six (14.3%) cases with systemic GvHD compared to only one (1.6%) case among those without systemic GvHD (p-value = 0.016). Those who developed ocular GvHD were older than those who did not develop the condition (13.5 versus 8.9 years, respectively; p-value = 0.015). All other factors analyzed were not associated with oGVHD (Table 4).

**Table 4:** Risk factors for ocular graft-versus-host disease (*n* = 105).

Risk factor	Ocular GvHD		p-value
	No (*n* = 98)	Yes (*n* = 7)	
Age, years – mean (SD)	8.9 (4.9)	13.5 (3.4)	0.015
Sex - n (%)			0.700
Male	58 (59.2)	5 (71.4)	
Female	40 (40.8)	2 (28.6)	
Diagnosis - n (%)			0.280
AAL	36 (36.7)	2 (28.6)	
AML	25 (25.5)	4 (57.1)	
AA	14 (14.3)	1 (14.3)	
Others	23 (23.5)	0 (0)	
Conditioning regiment - n (%)			0.547
Myeloablative	77 (78.6)	6 (85.7)	
Reduced intensity	21 (21.4)	1 (14.3)	
Radiotherapy conditioning - n (%)			0.711
No	47 (47.9)	4 (57.1)	
Yes	51 (52.0)	3 (42.9)	
Donor sex - n (%)			0.437
Male	50 (51.0)	2 (28.6)	
Female	48 (48.9)	5 (71.4)	
Source of graft - n (%)			0.603
Bone marrow	82 (88.2)	6 (85.7)	
Peripheral blood	11 (11.8)	1 (14.3)	
Type of transplant - n (%)			0.706
Related donor	52 (53.1)	3 (42.8)	
Unrelated donor	46 (46.9)	4 (57.2)	
HLA mismatch - n (%)			0.429
No	66 (67.3)	6 (85.7)	
Yes	32 (32.7)	1 (14.3)	
Sex mismatch - n (%)			0.463
No	44 (44.9)	2 (28.6)	
Yes	54 (55.1)	5 (71.4)	

SD, standard deviation; GvHD, graft-versus-host disease; ALL, acute lymphoblastic leukemia; AML, acute myeloid leukemia; AA, aplastic anemia.

## Discussion

The incidence and risk factors associated with the development of oGvHD were analyzed in a single-institution chart review over a five-year period. The incidence of oGvHD in allo-HSCT patients varies widely from 30 to 70%, partly due to different diagnostic criteria. Approximately 40–90% of patients with systemic chronic GvHD will develop ocular disease which is usually diagnosed within two years of chronic GvHD diagnosis [1,5,9,10,11].

Several risk factors have been associated to systemic GvHD and oGvHD in adult patients. Previous history of acute GvHD is known to be a strong risk factor for chronic GvHD development [12]. Other risk factors such as severity of systemic GvHD, use of peripheral blood stem cells, recipient age and donor-recipient sex mismatch have been shown to increase the risk of chronic GvHD [5,6,12,13]. However, these findings are mostly based on adult patients and rarely in pediatric populations. Recipient age has been identified as one of the major risk factors for chronic GvHD in several studies with younger patients being considered to have less risk [14, 15, 16, 17].

Kondo et al. reported a lower incidence of chronic GvHD in a pediatric population (22%) when compared to studies in adult populations. They also reported that older patients (>10 years) had an increased risk of chronic GvHD [14]. Locatelli et al. [18] reported a similar low incidence of chronic GvHD in children (24%). Furthermore, Eisner et al. [19] reported a low incidence (29%) of chronic GvHD and found that recipient age was an important risk factor in pediatric patients, although this was not confirmed by multivariate analysis. Recipient age is therefore an important risk factor for chronic GvHD in children. In addition to recipient age, Kondo et al.[14] reported that acute GvHD, malignant disease, and a female donor to male recipient were identified as significant risk factors for chronic GvHD. In several reports, a female donor to male recipient was recognized as a risk factor for chronic GvHD based on the alloimmunization of the female donor [15,17]. In childhood hematopoietic stem cell transplantation (HSCT), however, HLA-identical siblings are rarely alloimmunized, and alloimmunized female donors would be considered non-identical. Therefore, it is unclear whether alloimmunization affects the development of chronic GvHD in childhood HSCT [14].

Reports on incidence rate and risk factors for oGvHD are poorly documented in the pediatric age group. Bradfield et al. reported an incidence of oGvHD in three of 93 patients (3.2%) who underwent transplantation [11]. Fahnehjelm et al. reported DES in 37 of 60 children (61.7%) who had undergone HSCT. They also found that DES was more common in female patients with malignant diseases, in male patients who underwent HSCT at older ages, and in patients who were exposed to repeated high trough levels of cyclosporine (Cyclosporine capsules) for immunosuppression. There were no associations between prolonged corticosteroid treatment, irradiation treatment or chronic GvHD with DES in the mentioned study. As previously reported in other studies, they also demonstrated that older recipients presented higher risk of developing oGvHD after allo-HSCT [20].

Suh et al. assessed ocular findings in 104 pediatric bone marrow recipients in a two-year follow-up. DES was found in 12.5%, cataracts in 23%, and fundus complications in 13.5% of the patients [21]. Accordingly, Bradifield et al. reported ocular complications in 20.3% of children who underwent organ and bone marrow transplantation (BMT) [11]. Cataracts were the most common ocular complication following the use of systemic steroids. The reported prevalence of cataracts in children has varied from 8.5% to 80% [11,21].

A high prevalence of DES, ranging from 17 to 44%, has been reported in studies of adult BMT. These studies also found a strong association between DES and the occurrence of acute GvHD. Suh et al. [21] found a lower prevalence of DES (12.5%) in a pediatric population. They also demonstrated associations between the occurrences of acute and chronic GvHD and DES (77% and 48% of DES patients, respectively).

In this study the prevalence of oGvHD was 6.7%, a lower prevalence compared to the general population but similar to other pediatric studies. In the current series, older patients presented a higher risk for oGvHD. None of other risk factors analyzed such as conditioning regime, source of graft, sex mismatch or malignant disease were associated with the development of oGvHD.

A lower prevalence of DES may be explained by the retrospective nature of the study and the fact that the evaluation of DES was less comprehensive than in prospective studies on adults. Mild cases of DES in preverbal children are more difficult to diagnose since children express subjective symptoms less frequently than adults. Worse yet, young children are harder to examine using the fluorescein staining and Schirmer tests. That said, the prevalence of DES may have been underestimated in this study. Ng et al. reported that 51.7% of pediatric BMT patients had tear abnormalities; none of these patients had dry eye symptoms despite a third of them having patchy fluorescein staining of the cornea [22].

Management of chronic oGvHD is essentially the same as for other types of severe dry eye disease, but understanding the status of the patient's systemic GvHD can influence the ocular treatment strategy. Therefore, multidisciplinary assessments with an ophthalmologist and hematologist-oncologist are important [23].

The supportive care goals for oGvHD involve lubrication, control of tear evaporation, control of tear drainage, epithelial support and reduction of ocular surface inflammation. The treatment needs to be matched to the particular mix of symptoms of each patient; the individual's systemic medications also should be taken into account [23]. The first steps in reducing the symptoms of decreased tearing are preservative-free artificial tears and punctal occlusion with silicone plugs or cautery. In case of severe or persistent epithelial damage, autologous serum eyedrops, which contain many growth factors and vitamins that support the healing and integrity of the ocular surface, can be prescribed. Topical therapies, such as corticosteroid or cyclosporine (Cyclosporine capsules) drops, can be effective and optimization of these therapies is warranted. Ocular care also consists of photoprotection along with regular evaluations for infection, cataract formation, and increased intraocular pressure. Regional care may include ocular ointments, a humidified environment, occlusive eye wear, moisture chamber eyeglasses, or gas permeable scleral contact lens for symptomatic relief [24].

It is important to follow transplant patients closely with serial Schirmer tests to assess the degree of wetting and to intervene early at the onset of ocular involvement even prior to the evolution of symptoms [24]. The Schirmer test without anesthesia may be difficult to perform and it is not recommended in younger children; an ophthalmologist's input may be needed for objective scoring in these children [25]. For children who are old enough to tolerate the procedure, routine Schirmer evaluations should be done to monitor tear production [24]. Chronic oGvHD might impact on the quality of life of the pediatric population especially those who have not even reached economically active age.

There is a lack of studies focused in oGvHD in children. A few reports from the United States [11,21], Japan [14] and Italy [26] have already reported their experience but no report on this issue in Brazil was found.

A few limitations should be mentioned in the current study. Due to the retrospective nature of the analysis, it was not possible to establish baseline ophthalmic examinations prior to allo-HSCT and to determine the potential influence of the conditioning regimen on the ocular surface. Diagnosis of oGvHD was based on Schirmer's test or subjective symptoms according to the NIH criteria (2005). Decreased corneal sensitivity may underestimate changes of the ocular surface and allow false negatives results. Less comprehensive and not always reliable information in preverbal children may also underestimate oGvHD prevalence.

It is recommended that a baseline ocular profile of tear dynamics and ocular surface parameters should be conducted before allo-HSCT as well as regular ophthalmological examinations after the procedure, rather than relying exclusively on the NIH criteria. This might enable the use of measurable clinical and objective parameters to avoid false negative cases. That said, a prospective study setting with a baseline ocular surface work up before allo-HSCT and setting a disease (oGvHD) cutoff point using the metric parameters proposed by the International Chronic Ocular GvHD Consensus group [27] may provide more reliable data.

## Conclusion

Although with a relatively lower prevalence compared to adults, oGvHD with its morbidities is a concern after allo-HSCT in pediatric patients. Regular ophthalmologic assessments following allo-HSCT are therefore recommended for early detection and treatment of these potentially problematic complications in pediatric patients.

## Ethics

Ethics committee approval number: 014/11 – IOP, 0269/10 Federal University of São Paulo (UNIFESP).

## Contribution of the author

All authors contributed significantly to the work reported in this paper. Cinthia Kim contributed to conceptualization, methodology, data curation, formal analysis, writing of the original draft, and review and editing of the manuscript. Patricia Serapicos, Cintia Lustosa e Adriane Ibanez provided resources and assited with data curation. Falvio Hirai was responsible for conceptualization, methodology, formal analysis, and manucript review and editing. Victor Zecchin contributed to conceptualization, resources, and data curation. Lauro Oliveira led the conceptualization, methodology, investigation, supervision, review and editing of the manuscript, and secured funding. All authors reviewed and approved the final version of the manuscript.

## Conflicts of interest

The authors declare no conflict of interest.
